# On the Use of Collodion for Erections Accompanying Blennorrhagia

**Published:** 1853-08

**Authors:** 


### SURGERY.

*On the use of Collodion for Erections accompanying Blennorrhagia.* By Dr. Doringer.—In the *Med. Central Zeitung*, there is reported a case of a rather curious application of collodion for gonorrhœal erections, and the result was such as we would like to see borne out by other cases: A young man, aged 28, was attacked for the third time with a blennorrhagia, which was accompanied by such severe and painful erections, that the patient could hardly stay in bed half an hour. After having tried without avail both camphor and narcotics, Dr. Doringer ordered cold fomentations, and when the penis had resumed its natural size, the application over its whole extent, even including its prostatic portion, to a strong coating of collodion. This had the desired effect, for from that moment the patient had no erection, and suffered only from a slight scalding in passing urine. What proves that the amelioration was really due to the means employed is, that on the morrow, the collodion being taken off, the erections returned, but not so severely, and again ceased on the application of a fresh coating of collodion.—*Med. Times and Gaz.*, from *Rev. Med. Chir. de Paris.*

